# Severe cardiac valvular calcification in two Chinese brothers with mandibuloacral dysplasia type A: a case report

**DOI:** 10.3389/fcvm.2025.1657197

**Published:** 2025-11-20

**Authors:** Yi Guo, Xinyi Zhang, Yanfei Meng, Qiuzhe Guo, Xiaoqi Wang

**Affiliations:** 1 Department of SICU (Surgical intensive care unit), Fuwai Yunnan Hospital, Chinese Academy of Medical Sciences, Affiliated Cardiovascular Hospital of Kunming Medical University, Kunming, Yunnan, China; 2 Department of Ultrasound, Fuwai Yunnan Hospital, Chinese Academy of Medical Sciences, Affiliated Cardiovascular Hospital of Kunming Medical University, Kunming, Yunnan, China; 3 Department of Cardiac Surgery, Fuwai Yunnan Hospital, Chinese Academy of Medical Sciences, Affiliated Cardiovascular Hospital of Kunming Medical University, Kunming, Yunnan, China

**Keywords:** cardiac valvular calcification, progeroid syndromes, aortic stenosis, transcatheter aortic valve implantation (TAVI), laminopathies

## Abstract

Mandibuloacral dysplasia type A (MADA) is a rare progeroid syndrome associated with mutations in the Lamin A/C (LMNA) gene, primarily affecting skeletal, cutaneous, and adipose tissues. While certain LMNA gene mutations are known to cause cardiomyopathy and conduction system disease, severe early-onset calcific valvular heart disease is not conventionally considered a typical feature of MADA. This report describes two brothers from a consanguineous Han Chinese family who presented with classical MADA phenotypes alongside severe, early-onset cardiac valvular calcification. Genetic investigation revealed that both affected brothers carried a homozygous missense mutation, c.785A > G (p.Glu262Gly), in the LMNA gene. The elder brother, aged 41, successfully underwent transcatheter aortic valve implantation (TAVI) due to severe aortic valve stenosis. This finding represents the first association, to our knowledge, between the homozygous LMNA c.785A > G (p.Glu262Gly) mutation and significant severe early-onset cardiac valvular calcification manifesting as a prominent feature within the MADA phenotype, thus expanding the clinical spectrum associated with MADA and this specific LMNA variant. This case highlights a potentially underrecognized cardiovascular manifestation in MADA patients and underscores the importance of comprehensive cardiac assessment in affected individuals, particularly those from consanguineous families.

## 1 Introduction

Mandibuloacral dysplasia (MAD) is a rare, autosomal recessive disorder presenting with features suggestive of premature aging (progeria) (1). The clinical phenotype typically includes mandibular and clavicular hypoplasia, progressive acroosteolysis of distal phalanges, delayed cranial suture closure, cutaneous atrophy, and a characteristic pattern of partial lipodystrophy, primarily affecting the extremities while sparing the face and neck (2). Genetically, MAD is classified into two main subtypes: type A (MADA), associated with biallelic mutations in the *LMNA* gene, and type B (MADB), associated with mutations in the *ZMPSTE24* gene, which encodes a zinc metalloproteinase involved in prelamin A processing (3).

The *LMNA* gene encodes lamins A and C, type V intermediate filament proteins that are principal components of the nuclear lamina, a meshwork underlying the inner nuclear membrane (4). Lamins provide structural support to the nucleus, and anchor chromatin, and participate in crucial cellular processes including DNA replication, transcription regulation, and signal transduction (5). Mutations in *LMNA* give rise to a wide spectrum of human diseases collectively known as laminopathies, demonstrating remarkable tissue specificity despite the ubiquitous expression of lamins A/C (6). These disorders range from muscular dystrophies (e.g., Emery-Dreifuss muscular dystrophy), peripheral neuropathy (Charcot-Marie-Tooth disease type 2B1), lipodystrophy syndromes (e.g., Dunnigan-type familial partial lipodystrophy), and severe progeroid syndromes like Hutchinson-Gilford progeria syndrome (HGPS) and MADA itself (7).

While certain laminopathies, particularly those associated with specific *LMNA* mutations, frequently involve the cardiovascular system leading to dilated cardiomyopathy, conduction system disease, and atrial fibrillation (8), MADA is primarily recognized for its skeletal, cutaneous, and adipose tissue manifestations (8). Significant cardiac valvular pathology, specifically severe calcific aortic stenosis developing at a relatively young age, is not considered a typical feature of MADA and is infrequently reported in the literature. Valvular calcification is increasingly understood not as a passive degenerative process, but as an active, regulated pathobiological process sharing similarities with osteogenesis, potentially influenced by underlying genetic predispositions or systemic metabolic derangements that affect cellular function within the valve leaflets (9, 10).

Given the rarity of severe valvular disease in MADA, this report details the clinical course of two brothers from a consanguineous Han Chinese family who presented with classical MADA phenotypes alongside severe, early-onset cardiac valvular calcification. Genetic investigation revealed a homozygous missense mutation, c.785A > G (p.Glu262Gly), in the *LMNA* gene. We describe the diagnostic evaluation, and multidisciplinary treatment approach including transcatheter aortic valve implantation (TAVI) for the elder sibling, and discuss the potential implications of this specific *LMNA* mutation in contributing to this unusual cardiac phenotype within the context of MADA. This case highlights a potentially underrecognized cardiovascular manifestation associated with MADA and underscores the importance of comprehensive cardiac assessment in affected individuals.

## 2 Case presentation

Ethical approval was obtained from the Ethics Committee of Fuwai Yunnan Cardiovascular Hospital (IRB2017-BG-028), and informed consent was obtained from the patient and family members.

**Figure 1:**
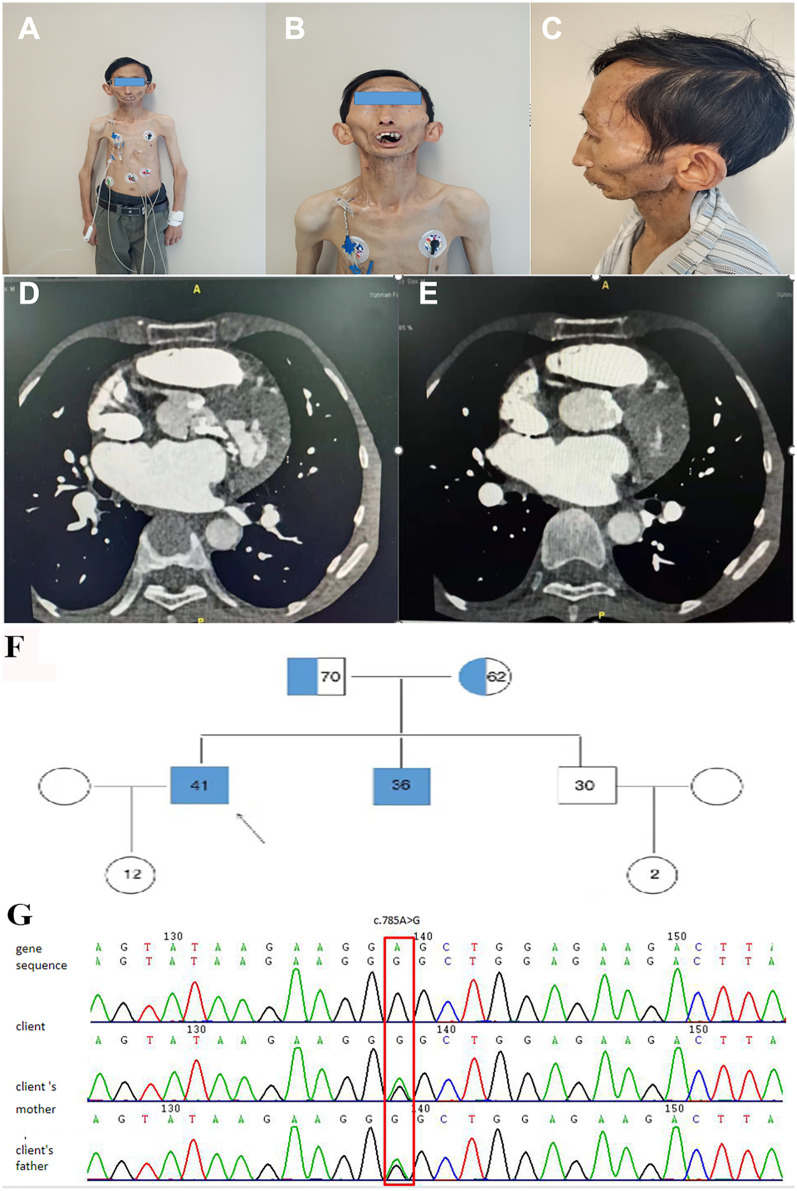
Clinical, radiological, and genetic findings. **(A–C)** Elder brother showing mandibular hypoplasia **(A)**, dental crowding **(B)**, loss of subcutaneous fat with prominent scalp veins and bulbous cheeks **(C)**, tapered nasal tip **(B)**, and sclerodermatous skin changes on trunk and upper limbs. **(D,E)** Cardiac CT revealing extensive calcification (white areas) in the aortic and mitral annuli, aortic valve leaflets, and left ventricular outflow tract. **(F)** Family pedigree indicating autosomal recessive inheritance; filled symbols: affected individuals (homozygous LMNA c.785A > G), half-filled: heterozygous carriers; arrow marks the proband. **(G)** Sanger sequencing confirming homozygous LMNA c.785A > G (p.Glu262Gly) in the proband and heterozygosity in a parent.

A 41-year-old Han Chinese male, the product of a consanguineous marriage, presented with exertional dyspnea and fatigue, symptoms attributable to severe aortic valve stenosis. His medical history was significant for features characteristic of MADA, diagnosed in childhood. Physical examination revealed a markedly short stature (height: 1.2 m) and low body weight (26.5 kg). Craniofacial features included mandibular hypoplasia, dental crowding, a tapered nasal tip, and prominent scalp veins (Figures 1A–C). There was generalized lipodystrophy with striking loss of subcutaneous fat in the limbs, contrasted with relative preservation in the face and neck, accompanied by thin, atrophic, sclerodermatous skin changes (Figures 1A–C). Despite these physical abnormalities and slowed growth during adolescence following a normal birth and early development, his cognitive function was normal, having completed college and pursued a career as a physics teacher.

**Figure 2:**
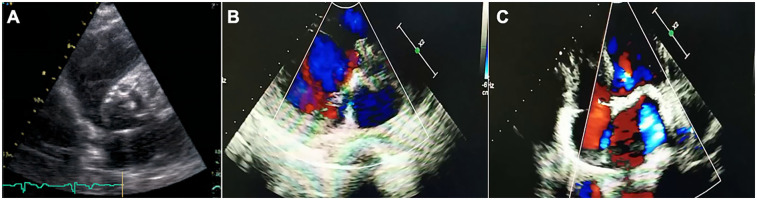
Transthoracic echocardiographic assessment of aortic stenosis in the proband. **(A)** Parasternal short-axis view showing tricuspid aortic valve with markedly thickened, calcified leaflets (arrows) and restricted systolic opening. **(B,C)** Apical five-chamber views with color Doppler demonstrating high-velocity, turbulent systolic flow (mosaic pattern) across the stenotic valve, consistent with severe obstruction.

He was diagnosed with severe aortic valve calcification and stenosis at age 39 during an investigation of his symptoms. A comprehensive cardiac assessment confirmed complex valvular heart disease. Transthoracic echocardiography showed a severely stenotic aortic valve, noted to be morphologically tricuspid but with extensive calcification, leaflet thickening, and commissural fusion restricting opening (Figure 2A). Color Doppler interrogation revealed high-velocity, turbulent flow consistent with severe stenosis (peak gradient > 80 mmHg, mean gradient > 50 mmHg, calculated valve area < 0.8 cm²), accompanied by moderate mitral stenosis with regurgitation, moderate tricuspid regurgitation, and markedly elevated estimated systolic pulmonary artery pressure (84 mmHg) (Figures 2B,C). Standard laboratory investigations, including complete blood count, inflammatory markers (CRP, ESR), renal and hepatic function panels, fasting glucose, HbA1c, and serum phosphocreatine kinase, were unremarkable. Chest radiography demonstrated cardiomegaly and a distinct pyriform (pear-shaped) thoracic configuration. Cardiac computed tomography (CT) provided detailed anatomical information, confirming massive calcification involving the aortic valve leaflets, aortic annulus, and mitral valve annulus, and extending into the left ventricular outflow tract (Figures 1D,E).

Evaluation for common etiologies of premature valvular stenosis was undertaken. The patient had no history or evidence of hypertension, diabetes mellitus, or hyperlipidemia; his lipid profile was within normal limits. Electrocardiogram showed left ventricular hypertrophy but no significant conduction abnormalities and coronary CT angiography excluded obstructive coronary artery disease. The tricuspid morphology identified on both echocardiography (Figure 2A) and CT effectively ruled out bicuspid aortic valve disease, a common cause of stenosis in younger adults. Furthermore, serum calcium, phosphate, parathyroid hormone (PTH), and 25-hydroxyvitamin D levels were all within the normal physiological range, making primary disorders of calcium homeostasis an unlikely explanation for the extensive calcification.

Family history revealed that the patient's younger brother, aged 36, exhibited a similar MADA phenotype and had also been diagnosed with cardiac valve calcification (moderate aortic stenosis) at age 28, although his condition was less advanced clinically. Their parents, who are first cousins, and another younger brother were phenotypically unaffected (Family pedigree, Figure 1F, Table 1).

**Table 1:** Clinical and genetic characteristics of the family.

Feature	Proband (II-1)	Affected sibling (II-2)	Father (I-1)	Mother (I-2)	Unaffected sibling (II-3)
Age (years)	41	36	Unaffected	Unaffected	Unaffected
Sex	Male	Male	Male	Female	Male
LMNA p.Glu262Gly	Homozygous	Homozygous	Heterozygous	Heterozygous	Wild-Type (assumed)
MADA Phenotype	Yes	Yes	No	No	No
Height (m)	1.2	N/A	Normal	Normal	Normal
Weight (kg)	26.5	N/A	Normal	Normal	Normal
Aortic Stenosis	Severe	Moderate	No	No	No
Peak/Mean Gradient (mmHg)	>80/>50	N/A	N/A	N/A	N/A
Aortic Valve Area (cm^2^)	<0.8	N/A	N/A	N/A	N/A
Mitral Stenosis	Moderate	N/A	No	No	No
PA Systolic Pressure (mmHg)	84	N/A	N/A	N/A	N/A
Key Labs (Lipids, Ca, P, PTH)	Normal	Unremarkable	Unremarkable	Unremarkable	Unremarkable

Given the strong familial component and syndromic features, genetic testing was pursued. Whole-exome sequencing (WES) followed by confirmatory Sanger sequencing was performed on blood samples from the proband, his affected brother, and their parents. This analysis identified a homozygous missense variant, c.785A > G, located in exon 4 of the *LMNA* gene (NM_170707.4) on chromosome 1q22 (Chr 1:156104741). This nucleotide change results in the substitution of glutamic acid with glycine at codon 262 (p.Glu262Gly). Both affected brothers carried this mutation in the homozygous state, while both phenotypically normal parents were heterozygous carriers (Figure 1G). Literature and database searches (GnomAD, ExAC, HGMD, ClinVar) indicated that while other *LMNA* mutations cause MADA, this specific homozygous p.Glu262Gly substitution had not been previously reported in association with severe cardiac valvular calcification. Analysis of WES data did not identify any potentially pathogenic copy number variations (CNVs) that could explain the phenotype.

Due to the severity of the aortic stenosis and debilitating symptoms, aortic valve intervention was deemed necessary for the 41-year-old proband. After a thorough evaluation by a multidisciplinary heart team, considering the increased potential surgical risks associated with his short stature, thoracic cage deformity, and the heavily calcified aortic root and mitral annulus, alongside the patient's strong preference for a less invasive procedure, the decision was made to proceed with transcatheter aortic valve implantation (TAVI). The TAVI procedure was successfully performed without major complications. The patient was followed for 12 months post-procedure. His symptoms of exertional dyspnea and fatigue improved significantly, improving from NYHA class III to class II. Repeat transthoracic echocardiography at 6 months showed a well-functioning prosthetic valve (mean gradient <10 mmHg, EOA > 1.5 cm^2^) and a notable reduction in estimated systolic pulmonary artery pressure to $45$ mmHg. No late complications, such as valve thrombosis or significant paravalvular leak, were observed during the follow-up period.

## 3 Discussion

Mandibuloacral dysplasia type A (MADA) represents a rare segment of the progeroid syndromes spectrum, stemming from mutations in the *LMNA* gene and primarily impacting skeletal, cutaneous, and adipose tissues (11). While cardiomyopathy and conduction system disease are recognized features of certain *LMNA* mutations (12), severe, early-onset calcific valvular heart disease is not conventionally considered part of the MADA phenotype (12, 13). This report presents compelling evidence from two affected brothers linking the homozygous *LMNA* missense mutation p.Glu262Gly (c.785A > G) to such severe valvular pathology.

The diagnostic workup in the proband systematically excluded more common causes of severe aortic stenosis in a relatively young adult. The congenital bicuspid aortic valve was ruled out based on clear tricuspid morphology on imaging (Figure 2A). Standard atherosclerotic risk factors were absent, and coronary arteries were free of significant disease. Rheumatic heart disease was unlikely given the valvular morphology and lack of supporting history. Furthermore, normal calcium and phosphate metabolism excluded systemic mineral balance disorders as the primary driver of extensive valvular calcification (14, 15). The co-segregation of the homozygous p.Glu262Gly *LMNA* mutation with the MADA phenotype and severe cardiac valvular disease in both affected siblings, born from consanguineous parents who are heterozygous carriers, strongly suggests an association between this genetic variant and the observed pathology.

The p.Glu262 residue is located within the coiled-coil rod domain of lamin A/C, which is essential for filament assembly and protein-protein interactions (16). While the precise mechanism linking the p.Glu262Gly substitution to valvular calcification remains to be elucidated, mutations in lamins can disrupt nuclear architecture, alter mechanotransduction, affect signaling pathways and influence cellular differentiation and senescence—processes potentially involved in the aberrant osteogenic differentiation observed in calcific valve disease. It is plausible that the altered lamin function creates a cellular environment within the valve interstitial cells that is permissive or even promotes premature calcification, perhaps as part of the accelerated aging process characteristic of MADA (12).

The use of TAVI in progeroid syndromes, particularly MADA, is exceptionally rare but has been previously reported. Stefanescu Schmidt et al. described a successful TAVR in a 31-year-old patient with MADA, highlighting similar challenges related to syndromic features and vascular access (17). Faria et al. also reported TAVI in a very young patient (20 years old) with MADA and severe aortic stenosis, further suggesting that transcatheter approaches are feasible in this high-risk population (18). Our case adds to this limited body of evidence, specifically linking the homozygous p.Glu262Gly LMNA mutation to the severe calcific aortic stenosis necessitating this intervention. The decision to opt for TAVI over surgical replacement was consistent with these prior reports, reflecting the technical challenges posed by the patient's syndromic features (e.g., short stature, thoracic deformity) and extensive calcification burden. Our case provides the specific genetic context (homozygous p.Glu262Gly *LMNA* mutation) for severe calcific aortic stenosis requiring TAVI in MADA. The decision to opt for TAVI over surgical replacement was carefully considered, reflecting the technical challenges posed by the patient's syndromic features and extensive calcification burden.

While the genetic evidence is strong, the consanguineous background necessitates acknowledging the limitation that other, undetected homozygous variants might contribute to the phenotype. Nevertheless, given the known role of *LMNA* in progeroid syndromes and the exclusion of other common causes, the p.Glu262Gly mutation stands as the most likely significant contributor to the severe valvular disease observed in this family.

In conclusion, this report describes the first association, to our knowledge, between the homozygous *LMNA* c.785A > G (p.Glu262Gly) mutation and severe, early-onset cardiac valvular calcification manifesting as a prominent feature within the MADA phenotype. This finding expands the clinical spectrum associated with MADA and this specific *LMNA* variant, highlighting that significant cardiovascular, particularly valvular, pathology can occur. Clinicians managing patients with MADA should be aware of this potential complication and consider comprehensive cardiac evaluation, especially in individuals from consanguineous families or those presenting with any cardiovascular symptoms. Further investigation into the functional consequences of the p.Glu262Gly mutation on valvular cell biology is warranted.

## 4 Limitations

The primary limitation of this report is that it establishes an association rather than direct causation. The family's consanguinity raises the possibility that other unidentified homozygous variants may contribute to the cardiac phenotype. Furthermore, mechanistic evidence is lacking, as functional studies, in silico modeling, or histopathological analysis of valve tissue were beyond the scope of this retrospective clinical report. A secondary limitation is the absence of detailed quantitative hemodynamic data for the moderate mitral stenosis from historical records, which precludes a precise evaluation of its contribution to the patient's severe pulmonary hypertension. Future *in vitro* functional validation is warranted to elucidate the mechanism.

## Data Availability

The original contributions presented in the study are included in the article/Supplementary Material, further inquiries can be directed to the corresponding author.
